# Reducing the risk of false discovery enabling identification of biologically significant genome-wide methylation status using the HumanMethylation450 array

**DOI:** 10.1186/1471-2164-15-51

**Published:** 2014-01-22

**Authors:** Haroon Naeem, Nicholas C Wong, Zac Chatterton, Matthew K H Hong, John S Pedersen, Niall M Corcoran, Christopher M Hovens, Geoff Macintyre

**Affiliations:** NICTA Victoria Research Laboratory, Department of Electrical and Electronic Engineering, The University of Melbourne, Parkville, Victoria 3010 Australia; Cancer, Disease and Developmental Epigenetics, Murdoch Childrens Research Institute, Royal Children’s Hospital, Department of Paediatrics, The University of Melbourne, Melbourne, Australia; Australian Prostate Cancer Research Centre Epworth, Richmond, Australia and Division of Urology, Department of Surgery, University of Melbourne, Royal Melbourne Hospital, Parkville, Australia; TissuPath Specialist Pathology, Mount Waverley, Victoria 3149, Melbourne, Australia; Faculty of Medicine, Nursing and Health Sciences, Monash University, Victoria, 3800 Australia; Ludwig Institute of Cancer Research, Olivia Newton John Cancer and Wellness Centre, Austin Hospital, Heidelberg, Victoria Australia; Department of Computing and Information Systems, Melbourne School of Engineering, The University of Melbourne, Melbourne, Victoria 3010 Australia

**Keywords:** HumanMethylation450K BeadChip, SNPs, INDELS, Repetitive regions of DNA, SNP arrays, HM450K bead array, Epigenome-wide association studies, EWAS, Cancer, Epigenetics

## Abstract

**Background:**

The Illumina HumanMethylation450 BeadChip (HM450K) measures the DNA methylation of 485,512 CpGs in the human genome. The technology relies on hybridization of genomic fragments to probes on the chip. However, certain genomic factors may compromise the ability to measure methylation using the array such as single nucleotide polymorphisms (SNPs), small insertions and deletions (INDELs), repetitive DNA, and regions with reduced genomic complexity. Currently, there is no clear method or pipeline for determining which of the probes on the HM450K bead array should be retained for subsequent analysis in light of these issues.

**Results:**

We comprehensively assessed the effects of SNPs, INDELs, repeats and bisulfite induced reduced genomic complexity by comparing HM450K bead array results with whole genome bisulfite sequencing. We determined which CpG probes provided accurate or noisy signals. From this, we derived a set of high-quality probes that provide unadulterated measurements of DNA methylation.

**Conclusions:**

Our method significantly reduces the risk of false discoveries when using the HM450K bead array, while maximising the power of the array to detect methylation status genome-wide. Additionally, we demonstrate the utility of our method through extraction of biologically relevant epigenetic changes in prostate cancer.

**Electronic supplementary material:**

The online version of this article (doi:10.1186/1471-2164-15-51) contains supplementary material, which is available to authorized users.

## Background

In humans, methylation occurs mainly in the context of cytosines followed by guanines (CpGs) [1]. Over 70% of CpG sites throughout the genome are methylated, however, CpG-rich regions (known as CpG-islands), found in approximately 60% of gene promoter regions, are usually unmethylated [2, 3]. DNA methylation is an important epigenetic mechanism used by cells to regulate gene expression and is essential for normal cell development [4, 5]. Aberrant DNA methylation patterns have been observed in various human diseases [6, 7], including cancer where hypermethylation of CpG-islands with resultant transcriptional silencing of tumour suppressor genes is recognized as a common mechanism for gene regulation [8, 9]. As such, the determination of genome-wide DNA methylation status plays a crucial role in improving our understanding of mechanisms of disease formation.

Several methods have been developed to detect the DNA methylation of cytosines distributed over the human genome. These include methylated DNA immunoprecipation sequencing (MeDIP-seq [10]), reduced representation bisulfite sequencing (RRBS [11]), methylated DNA captured by affinity purification (MethylCap-seq [12]), whole-genome bisulfite sequencing (WGBS) and the lower-resolution assays such as Infinium HumanMethylation27 (HM27K) array [13, 14] and Infinium HumanMethylation450 BeadChip (HM450K bead array; Illumina, Inc, CA, USA) [15]. Each of these methods has advantages and short comings when detecting differentially methylated regions in disease studies (see [13, 14, 16–19] for reviews). Choosing which technology to use is usually determined by cost, with array technologies providing a cheaper option, albeit at lower resolution. However, a recent study profiling regions of differential methylation across a range of human samples demonstrated that only a small fraction of CpGs across the genome vary in methylation status [15]. This means that whole-genome sequencing approaches may not be necessary to undertake comprehensive methylation profiling, suggesting great promise for continued use of array based approaches such as the HM27K and HM450K assays.

The HM450K array is a cost and time efficient technology that makes it possible to assess the methylation status of over 450,000 CpGs in the genome for large sample cohorts [15]. The array includes coverage of 96% of CpG Islands and CpG shores, 99% of RefSeq genes, 94% of loci present on HM27K bead array, and additional CpGs identified as variable from various WGBS methylation investigations [15].

To detect the methylation status at individual CpG loci, the Illumina Infinium assay relies on hybridization of bisulfite-converted DNA fragments to bead-bound probes [15]. Two probe types exist on the array, Infinium I and Infinium II. Infinium I type probes interrogate the methylation status of a CpG using the ratio between two probes that hybridize either the methylated or unmethylated DNA template flanking the CpG of interest. Infinium II type probes use a single probe with a single fluorescent tagged base ligation that is specific for the methylated or unmethylated states of the CpG of interest. The different chemistries of Infinium I and infinium II probes and the fact they interrogate different sets of CpG populations, means that the probe groups have different distributions of DNA methylation measurements on the HM450K bead array [20]. As such, several R packages with a range of normalization methods for the HM450K bead array have been developed to account for this difference [13, 21–25].

While recent reports have illustrated the accuracy and reproducibility of this platform [15, 26–28], many studies have also reported that probes on the array may produce erroneous results due to genomic factors other than methylation that affect hybridization or base ligation. For example, a number of studies have shown that a probe’s ability to measure accurate DNA methylation can be affected by SNPs at the interrogated CpG (20,879 probes in [29], 40,484 probes in [30], and 66,877 probes in [31]). It has also been suggested that SNPs within 10 bp of the interrogated CpG can affect probes (36,535 in [29]). Given that previous studies on gene expression arrays, which also rely on hybridization, have shown that single nucleotide polymorphisms (SNPs) and short insertions and deletions (INDELs) overlapping probe regions affect hybridization [32–34], SNPs are likely to impact our ability to measure methylation using the HM450K array. In addition to variants affecting methylation calling, a significant number of probes have been shown to map to multiple locations in the genome. Cross-reactivity of these regions can compromise true signal detection by the array and many studies have suggested removal of these probes from analysis (29,233 X chromosome probes [31] and 40,590 autosomal probes [29]). This effect is confounded, since bisulfite treatment of DNA converts unmethylated C to T, rendering the “bisulfite genome” with reduced complexity, which facilitating more multiply mapped probes. It has also been suggested that probes which span regions in the genome containing repeats yield erroneous methylation calls [13, 35] and should be filtered. Probe filtering has even occurred in regions of copy-number change [36], in spite of a study that analyzed the effect of copy number on observed methylation at CpG sites using the HM27K array, and concluded that there was no systematic copy number effect on methylation status [37].

In many of these previous studies, the effects of factors causing noise in methylation measurement was not determined directly but inferred through observed trends such as increased standard deviation in probes affected by SNPs at the CpG across multiple samples from the same tissue [29]. Usually, in absence of more information, a conservative approach has been taken, aggressively filtering any probe which may be potentially affected. None of the previous studies have performed a systematic analysis of all of the potential factors affecting probes on the HM450K bead array. In this study, we perform a rigorous analysis of the effects of SNPs, INDELs, repeats and multi-mapping probes. In contrast to previous studies, we have compared these confounding effects against WGBS data. Our analysis yields a set of probes which should be removed during analysis as we have shown they provide a noisy signal (i.e. increased deviation in measurement using the HM450K bead array compared to whole-genome bisulfite sequencing). By removing these probes, we show recovery of biologically relevant results which would have been missed without our probe filtering approach.

## Results and discussion

Design of an array based technology which interrogates a large number of CpGs across the human genome is a difficult task. A comprehensive set of interrogation sites will inevitably contain probes which will be affected by SNPs, INDELs, multiple location hybridization and repeat sequences. The HM450K bead array contains a large number of interrogated CpG sites and it is up to users to decide which probes on the array they are willing to include in their analysis in light of the knowledge that some probes may provide a potentially ‘noisy’ signal. This becomes problematic when each study using this platform excludes their own panel of probes leading to apparently discrepant results. To enable users of this technology to make a more informed decision on which probes to include in their analysis, we performed a comprehensive analysis of the effects of SNPs, INDELs, repeats and genomic complexity, on the ability to measure DNA methylation status. To do this, we used four datasets:

1. HM450K profiling in H1-hESC cells with matched whole-genome bisulfite sequencing (WGBS) from the ENCODE project [38]. In this case, we used the WGBS results as a ‘gold-standard’ to determine the accuracy of selected probes on the array.
2. HM450K profiling of 63 cell lines from the ENCODE project. In this case we detected trends in probe readouts that were present across a large numbers of cell types.
3. HM450K profiling of primary prostate cancer samples. We used these data to show that noisy probes can affect the overall ability to detect meaningful biology.
4. HM450K profiling of 265 blood samples from Price et al. [29]. These data were used to show a similar trend to that observed in the original paper, that noisy probes show greater within tissue methylation variance than non-noisy probes.

Through analysis of each dataset we were able to identify a set of probes which were likely to provide a confounding signal. Removal of these probes resulted in a final collection of probes in which users can be confident in their measurements of DNA methylation status. While we provide a recommended set of probes to remove from analyses, it is ultimately up to the end user to choose a final probe set that best suits their expectations.

As two different chemistries are used for the Infinium I and Infinium II probes, we analyze them as independent probe groups as they may be affected differently. Table 1 provides information on the number of probes that are potentially affected by different genomic factors. If an ultra conservative approach to probe filtering was adopted, removing any probe potentially affected by other genomic factors, it can be seen from Table 1 that only 172,587 probes would remain for further analysis. While some factors may drastically affect the resulting methylation calls such as SNPs at the interrogated CpG, some phenomena such as SNPs in the probe body may have little effect on measured methylation state. As such, we believed it was possible to increase the set of high-quality probes by identifying those unlikely to be affected by certain phenomena. The following section outlines our decision making approach for inclusion or exclusion of certain probes based on comparison of the HM450K bead array with matched whole-genome bisulfite sequencing.

**Table 1:** **Summary of the probes which are identified as having hybridization problems due to multimapping, SNPs, repeats, INDELs and unknown factors (note: some probes may belong to more than one category)**

	Infinium I	Infinium II	Total
**Total number of probes on array**	**135,476**	**350,036**	**485,512**
Probes which map to multiple genomic locations	5,971	13,863	19,834
Probes containing only known INDELs	1,935	4,348	6,283
Probes containing known repeat regions	8,438	30,305	38,743
Probes which have a SNP/INDEL at interrogated CpG	12,746	57,372	70,118
Probes containing known SNPs	52,175	117,342	169,517
Probes affected by unknown factors^#^	1,394	13,840	15,440
**Total number of high-quality probes on array**	**52,817**	**119,770**	**172,587**
**Total number of potentially ‘noisy’ probes**	**82,659**	**230,266**	**312,925**

^#^ ’Probes affected by unknown factors’ are considered as probes which have absolute beta difference between WGBS and HM450K bead array greater than 0.3.

### Using a comparison of methylation status measured using the HM450K bead array against whole-genome bisulfite sequencing to determine ‘noisy’ probes

**Figure 1:**
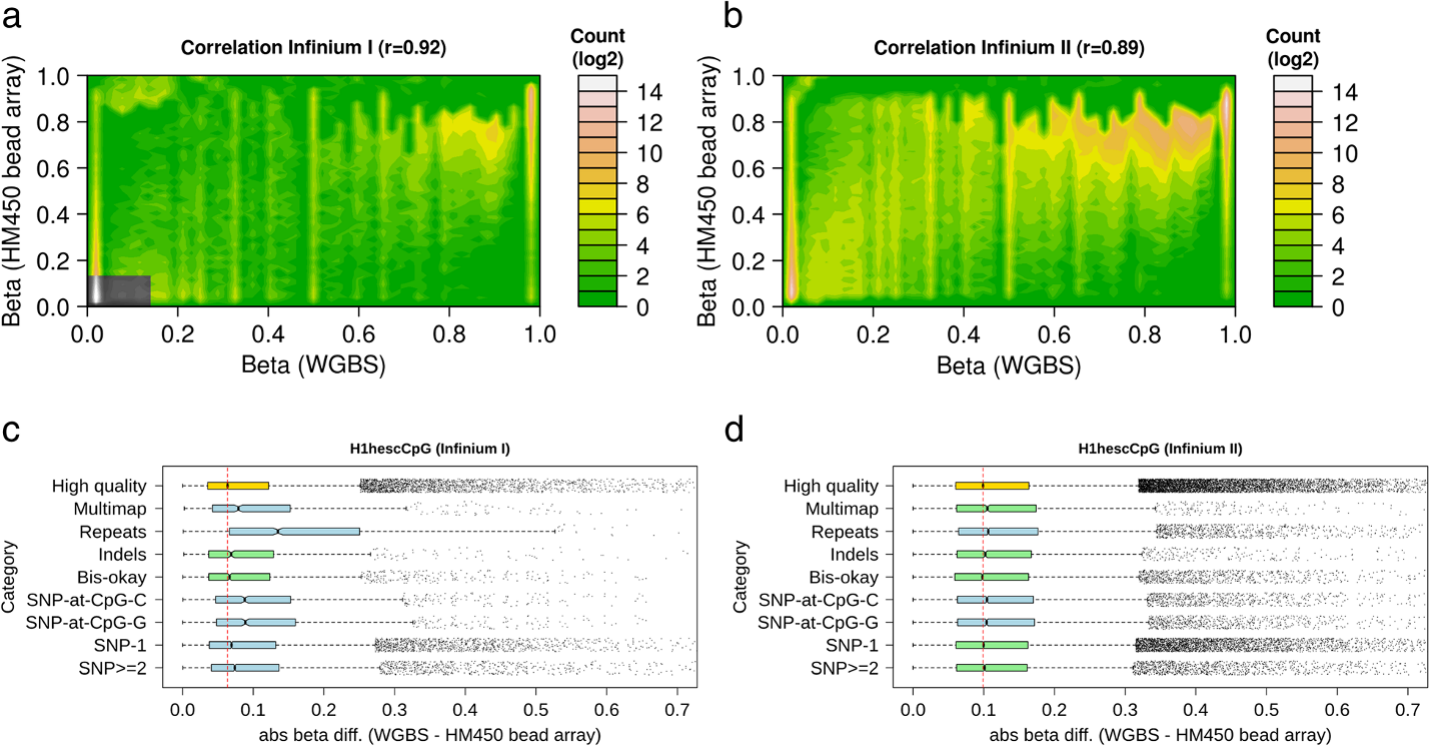
**This figure summarises the comparison between WGBS and HM450K beta-values for the H1-hESC cell line.** Figures **a)** and **b)** show contour plots demonstrating the correlation of DNA methylation between WGBS and HM450K bead array for Infinium I probes and Infinium II probes respectively. The contours (different colour intensities) capture the density of beta-values. It can be seen that most points reside close to 0, 0 and 1, 1, resulting in high-correlation between the platforms. Figures **c)** and **d)** show boxplots of DNA methylation results (absolute beta differences) between WGBS and HM450K bead array, plotted for different potential filtering categories for Infinium I and II probes respectively. The box extends from the first to the third quartile and whiskers extend to 1.5 times the interquartile range. Points outside this are considered outliers. The blue boxplots show the distribution of filtering category probes that were statistically significantly different from the high quality probes (golden boxplot) (P < 0.001) otherwise, the category is plotted as light green. The red dotted line depicts the median of a high quality probe set. Category definitions: High-quality - represents probes which are not affected by any genomic factors; Repeats - describes probes which hybridize to repetitive regions; Bis-okay – are probes which hybridize regions containing any C– > T SNP or T- > C SNP and are ‘okay’ in bisulfite space; SNP-at-CpG-C and SNP-at-CpG-G - are probes which have SNPs at the interrogated C and its neighbouring G position, respectively; Indels - are probes which hybridize regions containing INDELs; Multimap - are probes which hybridize to multiple genomic loci; SNP-1 - are probes which contain only a single SNP anywhere in the body; and SNP > = 2 - are probes which contain at least 2 SNPs anywhere in the probe body.

We compared methylation (beta values) from the HM450K bead array with those derived from whole-genome bisulfite sequencing, matched for genomic location and cell type. Overall the beta-values determined using the HM450K bead array showed high correlation with methylation determined using whole-genome bisulfite sequencing (r = 0.92 Infinium I, r = 0.89 Infinium II, Figure 1a, b). However, when we considered only the probes which were not potentially affected by any other genomic factor (Table 2), the correlation increased to r = 0.95 and r = 0.92 respectively (referred to from now on as high-quality probes). This suggested that the remaining probes could be affected by genomic factors other than methylation. However, before being able to determine which probes provided a noisy signal, it was necessary to estimate the expected background noise when comparing methylation measured using array hybridization to methylation measured using bisulfite sequencing.

**Table 2:** **Pearson correlation of DNA methylation results (beta-values) between WGBS and HM450K bead array for the H1-hESC cell line, measured for different categories**

	Infinium I				Infinium II			
Category	Unique probes	Median	Pearson corr.	P-value	Unique probes	Median	Pearson corr.	P-value
High quality	25,199	0.06	0.95	NA	83,565	0.10	0.92	NA
Multimap map	917	0.08	0.93	3.43e-09	2,602	0.11	0.91	3.72e-03
Repeats	1,645	0.13	0.85	6.1e-141	11,026\	0.11	0.79	9.03e-11
Indels	901	0.07	0.94	6.49e-02	2,993	0.10	0.91	8.32e-02
Bis-okay	2,734	0.07	0.95	7.74e-02	11,804	0.10	0.92	8.32e-02
SNP-at-CpG-C	1,074	0.09	0.93	7.07e-18	8,099	0.10	0.83	6.59e-05
SNP-at-CpG-G	1032	0.09	0.91	2.38e-21	7,488	0.10	0.82	2.42e-04
SNP-1	14,155	0.07	0.95	6.33e-12	40,426	0.10	0.91	2.30e-01
SNP > =2	7,067	0.07	0.94	2.36e-27	13,822	0.10	0.90	2.27e-01

Each probe count is the number of probes unique to each category and contains only probes which appear on the array and have a matching WGBS beta value. Category definitions: **High-quality -** contains probes which are not affected by any genomic factors; **Repeats -** describes probes which hybridize to repetitive regions**; Bis-okay –** are probes which hybridize regions containing any C- > T SNP or T- > C SNP and **are ‘**okay**’** in bisulfite space**; SNP-at-CpG-C and SNP-at-CpG-G -** are probes which have SNPs at the interrogated C and its neighbouring G position, respectively**; Indels -** are probes which hybridize regions containing INDELs**; Multimap - are** probes which hybridize to multiple genomic loci**; SNP-1 -** are probes which contain only *a* single SNP anywhere in the body**;** and **SNP > =2 -** are probes which contain at least 2 SNPs anywhere in the probe body.The p-values represent the difference in beta-value distribution calculated using the Wilcox-rank sum test [39] and multiple tested corrected using the Benjamini-Hochberg method [40]. The median of the absolute difference between WGBS and HM450K bead array of each set is also shown.

Array based methylation measurements are derived from continuous fluorescence intensities that are transformed into beta values ranging from 0 to 1 (unmethylated to methylated). Whereas sequencing based methylation measurements are derived from discrete read counts which are transformed to beta values ranging from 0 to 1. The comparison between the two beta values is somewhat analogous to comparing an analog to a digital measurement. Therefore, when comparing beta values from the two technologies there will likely be a background error rate which is due in a large part to the difference in measurement methods. We calculated the distribution of this background error rate in terms of absolute beta value difference between the sequencing derived beta value and the array derived for all high-quality probes (Figure 1c and 1d). There was a median difference in beta-value of 0.06 for Infinium I probes, and 0.10 for Infinium II probes between the array and sequencing methodologies. We used these high-quality probes as a gold-standard to assess the performance of other probes. To explore how different genomic factors affected array based measurements we observed if the difference in beta-value significantly increased above the expected background difference derived from the high-quality probes (see following sections). If so, we concluded that the probe set be removed from further analysis as the probes provided a ‘noisy’ signal.

For the remainder of this paper we consider differences in beta values between WGBS and the HM450K in terms of absolute differences. However, for completeness we have included Figure S10 in additional file 1 that illustrates the signed beta differences between WGBS and HM450K bead array. This figure shows that Infinium I probes on average tend to underestimate the methylation signal output by WGBS, while the Infinium II probes tend to overestimate the methylation status. The differences observed between the two probe types is likely due to the different genomic regions being interrogated. Infinium I probes interrogate CG rich sequences, and promoters, which are more likely to provide a robust methylation signal, albeit more variable. Whereas Infinium II probes interrogate additional regions such as gene bodies and intergenic regions. These regions are more difficult to profile and are more likely to be affected by repeats resulting in a higher background error rate plus less variability due to factors other than differential methylation.

#### Probes which hybridize multiple genomic locations

In the case of probes which hybridize to multiple locations in the genome, it is difficult to determine which genomic region gives rise to the measured methylation state. Therefore, without individual analysis of each probe and its possible hybridizable regions, including those probes in an array wide analysis is potentially problematic. We estimated a total of 19,834 probes fall into this category. When compared to WGBS, there was a median difference in beta-value of 0.08 for Infinium I and 0.11 for Infinium II (Figure 1c and 1d, and Table 2 category Multimap) and a correlation of 0.93 and 0.91 respectively (Table 2 category Multimap). The difference in beta-value distribution was significantly higher than background in the case of Infinium I probes (p < 0.001). Given this error rate, and the difficulty in determining which genomic region gives rise to the observed methylation status we recommend removal of these probes from subsequent analysis for both Infinium I and II probes. If these probes were to be used for further analysis, they would have to undergo a deconvolution process to determine which genomic region (s) give rise to the observed methylation status.

#### Probes which hybridize repetitive regions

Probes which hybridize to repetitive regions have the potential to encounter unusual hybridization issues. The repeat may be small and occur multiple times in the probe, in which case the probe may align at multiple locations. Or the repeat may be large and span the entire probe, therefore the probe can potentially hybridize to multiple locations. Furthermore, repeat families can be highly polymorphic introducing another layer of confounding factors. We determined a total of 38,743 probes fall into this category. These probes had the highest median difference in beta value at 0.11 for Infinium I and 0.13 for Infinium II, both of which were significantly different to the expected background difference (p < 0.001) when compared to WGBS (Figure 1c and 1d, and Table 2 category Repeats). Given this high observed difference in beta value and the fact that the effects of repeats on hybridization are relatively unexplored, we recommend a conservative approach be taken and these probes be removed from further analysis.

#### Probes which hybridize regions containing INDELs

The prevalence of INDELs across the genome has recently shown to be significant [41]. Therefore INDELs may have a considerable effect on probe hybridization and observed methylation status. A total of 6,283 probes hybridize to regions potentially containing INDELs. However, the median difference in beta value of these probes (0.07 Infinium I, 0.10 Infinium II, Figure 1c, d, Table 2 category Indels) was not different from the expected background difference and the correlation was similar to the high-quality probes (Table 2 category High quality). This may be due to the fact that while many INDELs have been identified and annotated, they are not frequent enough in any given sample to have an effect. By default we recommend including probes which are annotated with INDELs in subsequent analyses. However, this is highly dependent on how the HM450K platform is being used. For population-wide studies removal of these probes is prudent in absence of any genotyping data to determine exactly which probes will be affected. While if an experiment is investigating in an *in vitro* cell line system, it is plausible to include these probes in the study given that the cell lines are derived from the same parental line.

#### Probes which hybridize regions containing SNPs

For probes that hybridize regions containing known SNPs it is not immediately obvious whether the SNP will have an effect on measured methylation status. For instance, as each probe is hybridizing a bisulfite treated genome, any C– > T SNP or T- > C SNP which occurs outside a CpG is likely to always be a T after bisulfite conversion unless it is part of a CpG site within the probe. Therefore SNPs of this nature should have no affect on hybridization. We observed this to be the case for both Infinium I and Infinium II probes where the difference in beta value and correlation were the same as that of the high-quality probes (Table 2, category Bis-okay). We therefore recommend including probes annotated with these SNPs in subsequent analyses for both Infinium I and II probes (total 14,538).

**Figure 2:**
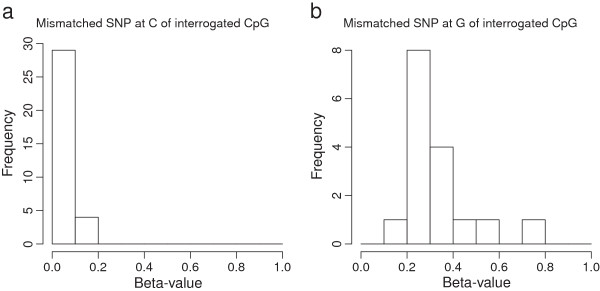
**This figure shows a histogram of the number of probes with SNPs at the interrogated CpG (y-axis) and their beta-value (x-axis).** In each case, these probes have been shown to have a SNP which causes a mis-match in the probe sequence at the C of the interrogated CpG **(a)** and at the G of the interrogated CpG **(b)**. These plots were generated using HM450K data from the H1-hESC cell line.

Of the remaining probes which hybridize regions containing a SNP, those which have SNPs at the interrogated CpG are expected to have a large effect on the resulting methylation measurement. A base change at the interrogated CpG would cause an otherwise methylated site to be considered unmethylated. To observe if this was the case, we used genotyping information for the H1-hESC cell to find all interrogated CpG locations where the C was in fact disrupted. All of the probes which had a homozygous SNP at the interrogated C (Figure 2a) showed no methylation. Similarly, nearly all of the probes with a homozygous SNP at the neighboring G also showed no methylation (Figure 2b). This would suggest that SNPs at these locations can have a dramatic effect on methylation and these probes should be removed from analysis. In the broader case where an interrogated CpG has an annotated SNP (but we do not know the genotype), our comparison with bisulfite sequencing data showed there was a significant increase in absolute beta value difference for SNPs at both locations for both Infinium I and Infinium II (Figure 1c and 1d, and Table 2 categories SNP-at-CpG-C and SNP-at-CpG-G). However, in the case of Infinium II, this difference was not highly significant suggesting that this approach may be too conservative. We use ENCODE data to justify why this may be the case in the following section. In spite of this, by default we opt to remove these probes from subsequent analysis. However, we recommend that analyses be carried out with and without these probes for completeness. If genotyping information is available for the sample being studied, this can be used to determine which probes should be included or discarded.

Finally, we looked at the effect of SNPs when they reside in the probe body. Koboldt et al. [35] have suggested that probes overlapping SNPs within 10 bp of the interrogated CpG affect the ability to measure DNA methylation, and recommend removing them from subsequent analyses. However, when we looked at the difference in beta value for probe sets with SNPs at different locations in the probe body, we saw no such bias (Figure S2 in Additional file 1). Instead, we analyzed the overall effect of a single SNP, and 2 or more SNPs anywhere in the probe body. For Infinium I probes our comparison with WGBS data showed that even a single SNP in the probe body resulted in a significant increase in absolute beta-value difference (Figure 1c and 1d, and Table 2 categories SNP-1 and SNP > =2). However, Infinium II probes seemed to tolerate 2 or more SNPs in the probe body. This is likely due to the difference in design between the two probes. Infinium II probes have a degenerate base at each CpG in the body which allows the hybridization of C or T, whereas Infinium I probes have an exact match [15]. Therefore, we recommend that Infinium I probes with overlapping SNPs in the probe body be removed from subsequent analyses, whereas Infinium II probes could be kept. However, it is important to note that in this case, if there is a mismatch, a smaller pool of templates would be contributing to the intensity signal of the probe. If genotyping information is available, it is possible to determine exactly which probes should be kept or discarded.

#### Using average heterozygosity to rescue probes

It has been suggested that SNPs with low average heterozygosity measurements are less likely to have an effect on probe hybridization compared to those with high average heterozygosity [29]. We measured the absolute difference in beta-value for probe sets with SNPs of varying average heterozygosity (Figure S3 in Additional file 1). For Infinium I probes, even SNPs with low average heterozygosity had a significant effect on the ability to accurately measure methylation status (Figure S3a in Additional file 1). For Infinium II probes, probes with SNPs with average heterozygosity between 0.2 and 0.3 and greater than 0.4 appeared to have a significantly larger difference in beta value, whereas those outside these ranges showed potential for providing a way to ignore these SNPs. However, overall, using average heterozygosity did not appear to provide any significant improvement. This is likely due to the fact that the population of individuals used to determine average heterozygosity (1000 genomes project [42]) is not sufficiently matched to the data we have used for comparison (H1-hESC cells). Therefore we conclude that average heterozygosity is only useful for ignoring the effect of certain SNPs when estimated from a closely matched population of individuals to the cell being studied.

#### Probes which hybridize regions affected by unknown factors

In the previous sections we identified and tested a range of genomic factors that may affect probes on the HM450K array. However, there may be other factors which we have not considered which cause a probe to output an erroneous result. Probes in this category are likely to produce beta values with large discrepancies compared to WGBS beta values. Figure S9 in Additional file 1 shows that some of the probes (5,126 Infinium I and 20,732 Infinium II) hold beta differences between WGBS and HM450K bead array greater than 0.3. We deem these probes to be affected by some unknown genomic factors and hence recommended they be removed from the analysis pipeline. Further analysis shows that 1,394 out of 5,126 Infinium I and 13,840 out of 20,732 Infinium II probes are unique to this category (Table 1). Please note, these probes were considered erroneous in light of data from a single cell-line. To improve estimates on the beta-value difference, additional comparisons are required to ensure only probes that show a consistently large deviation in beta-value are removed.

#### Probe filtering summary

Table 3 provides a summary of the probes we recommend to be discarded or kept in light of our comparison between HM450K and WGBS profiling of H1-hESC cells. An aggressive filtering procedure would remove 64% of probes (312,925) on the array (Table 1), however, our analysis has demonstrated that some of these probes do not provide a ‘noisy’ signal and can therefore be ‘rescued’. This results in a filtering procedure which removes only 39% of probes (190,672) on the array. We reiterate that some of the filtering steps we have used can be altered from the default depending on the type of study being performed. We provide an overview of the filtering procedure (Additional file 1: Figure S8) and filtering annotations (Additional files 2 and 3) for this purpose.

**Table 3:** **Summary of the probes which are removed or kept for further analysis**

	Infinium I	Infinium II	Total kept
Total number of probes on array	135,476	350,036	-
Probes which map to multiple genomic locations	1,581	3,728	0
Probes containing known repeat regions	2,845	13,673	0
Probes containing known INDELs	1,885	4,101	5,986
Probes containing SNP and interrogated CpG	9,568	42,800	0
Probes containing 1 SNP in the body	26,700	72,315	77,545*
Probes containing > =2 SNPs in the body	20,043	34,330	34,675*
Probes affected by unknown factors	1,394	13,840	0
Probes affected by multiple factors	16,857	49,580	4,047*
Total number of ‘rescued probes’	7,367	114,886	122,253
**Total number of high-quality probes**	**52,817**	**119,770**	**172,587**
**Total number of probes kept for further analysis**	**60,184**	**234,656**	**294,840**

*Some of these probes were rescued due to being bisulfite OK.

The probe counts depicted in this table are those which are unique to each category. Any probes which have are affected by multiple factors appear in the ‘Probes affected by multiple factors’ category.

### Beta-value trends across HM450K profiling of 63 ENCODE samples

It is known that methylated cytosines have a higher rate of deamination [43]. Thus cytosines which are frequently methylated will have a greater chance of having a SNP annotated at the same location [44, 45]. This is reflected by the fact that there are total of 70,118 probes which have an annotated SNP at the interrogated CpG, the second largest of all of the categories (Table 1). While many previous approaches have removed these probes en masse [36, 46], we argue that they could remain for subsequent analysis. This is due to a number of reasons: firstly, by looking at the methylation profiles of 53 cell lines from the ENCODE project [38], we observed that the majority of probes with annotated SNPs at the CpG have a high-beta value (Figure S1 in Additional file 1). From this, we can infer that on average, most of these probes are not in fact affected by SNPs. Furthermore, while a SNP in the probe body may affect the methylation readout for the CpG, a SNP at the interrogated CpG is affecting the methylation status of that exact location in the genome that is being interrogated. Therefore, unless the analysis is specifically avoiding cases where a SNP is causing a region to be unmethylated, the 70,118 probes could be considered for further analysis. While by default we have opted to remove these probes, if the study being undertaken involves a genetically homogeneous population of cells (including cell lines), it would be worth considering including these probes in the analysis.

### The effect of probe filtering: A case study of methylated regions in prostate cancer

In order to examine the impact our probe filtering efforts might have on the analysis of real-world primary data, we analyzed clinical prostate cancer specimens. The methylation status of prostate cancer tissues is of particular interest as recent studies show that methylation changes are a key driver of tumour progression [47]. In addition, results from WGBS of prostate tumours indicate that the majority of regions showing differential methylation reside outside CpG islands and that the vast majority of changes in CpG methylation status are not correlated with changes in gene expression [48]. Interestingly, inhibition of RNA expression did not inversely correlate with CpG island methylation status suggesting that arrays that focus on CpG island probes, as exemplified by the 27 K array, will be deficient for a proper analysis of methylation changes in prostate cancer. Therefore, it is important to be able to detect accurate methylation changes using the HM450K platform.

To explore this, we profiled four prostate tumour tissues and four benign prostate tissues using the HM450K bead array. These samples were used to observe the effects of probe filtering on downstream analysis. We took a typical analysis approach, observing differential probes between the two sets of samples (tumour versus benign). Using minfi with SWAN normalization, we determined the list of significantly differentially methylated probes between tumour and benign tissue with, and without probe filtering.

Without probe filtering, 45,376 probes were determined differentially methylated (multiple tested corrected p-value < 0.05). With filtering, 42,132 probes were differentially methylated. 30,439 probes were common between the lists, with 14,937 probes being unique to the unfiltered list and 11,693 probes being unique to the filtered list of differentially methylated probes. When these probes were mapped to gene promoters, the unfiltered approach yielded 891 unique differentially methylated genes and the filtered approach 698 unique differentially methylated genes.

**Figure 3:**
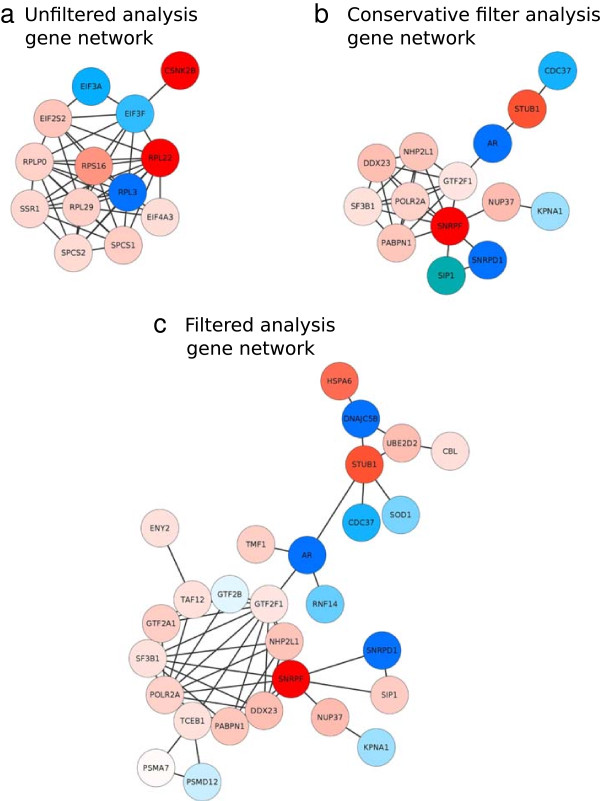
**This figure represents gene interaction networks from the string protein-protein interaction database.** The networks were derived from 3 sets of differentially methylated gene promoters: **(a)** the genes uniquely identified with no probe filtering, **(b)** with conservative probe filtering, and **(c)** our recommended probe filtering procedure. Dark blue represents unmethylated gene promoter in prostate cancer and red represents methylated gene promoters in prostate cancer.

To explore the biology behind each of these gene lists, we input each gene list into the String Protein-Protein Interaction database to determine which genes were known to interact with each other (see Method section Gene network construction for details). The unfiltered gene set indicated that the most connected gene network represented genes mostly involved in the ribosome which is not particularly associated with prostate cancer (Figure 3a). In stark contrast, the filtered gene list yielded a connected network with androgen receptor (AR) at the centre (Figure 3c). AR is a clinically confirmed key player in the progression of prostate cancer as blockade of androgens by surgical or chemical means has formed a vital part of clinical management for many decades [49]. As prostate cancers inevitably become resistant to androgen deprivation therapy, the molecular underpinnings of AR, its effects, regulation and interactions within pathways, remains a central theme in contemporary prostate cancer research with respect to therapy and biology [50, 51]. Using our filtering approach, we were able to observe the decreased methylation of the AR promoter in tumour tissue compared with benign prostate gland samples. Assuming a corresponding increased transcription of the AR gene, this would be consistent with its described biology in prostate cancer [52, 53]. Importantly, this network would have been overlooked in the absence of our probe filtering procedure and emphasizes the need for careful consideration of confounding genomic phenomena on probe effectiveness and accuracy. Furthermore, if an overly conservative approach to filtering is adopted (removing all probes potentially affected by different phenomena), this extensive network signature is similarly unobservable (Figure 3b).

### High-quality versus noisy probes: a case study of blood samples

**Figure 4:**
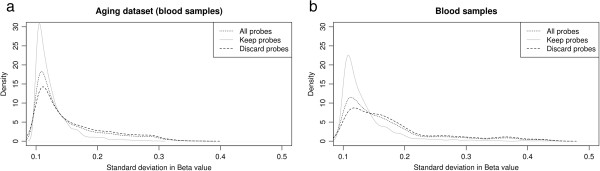
**This figure shows the distribution of standard deviation in beta values for probes on HM450K bead array, using (a) 4 blood samples and (b) 261 blood samples.** In each case, the distribution of standard deviation in beta values (> = 0.10) was plotted for all probes, for probes kept for subsequent analysis (Keep probes), and for the recommended removal of probes (Discard probes).

Theoretically, probes which are affected by various genomic factors would result in a pattern of high within-tissue standard deviation (SD) in beta values [29]. To test the applicability of our filtering procedure in light of this, we compared the distribution of SD in beta-values for different sets of probes: all probes (ALL), probes which are recommended by us to be removed prior to data analysis (DISCARD), and the probes which are kept for the analysis (KEEP). This comparison was done using two datasets: a set of 4 blood samples from [29], and a larger blood sample dataset of 261 individuals aged in the range of 19 to 61 from [54] (see method section for details). A comparison of the SD distributions for the 4 blood samples shows that the high-quality KEEP set of probes shows significantly lower standard deviations in beta-value than the DISCARD set of noisy probes (p-value = 1.97e-84) based on Wilcox-test [39]. Figure 4a illustrates this pattern by a shift in the density curve for SD in beta values for probes annotated with ALL, KEEP and DISCARD. Similar results were also obtained for the larger 261 aging dataset (see Figure 4b for illustration). By removing ‘noisy’ probes from the analysis, within tissue standard deviation is decreased, facility more powerful downstream comparisons of differential methylation between tissues.

### Exploring the filtered probe set: what is lost?

In the previous sections, we provided a principled way to reduce noise in the HM450K bead array via removal of probes which were affected by certain genomic phenomena.

By using a direct comparison with whole-genome bisulfite sequencing data, only the probes showing a noisy signal were removed thereby maximizing the number of probes retained for subsequent analysis. We suggested the removal of 190,672 (75,292 Infinium I and 115,380 Infinium II) (39%) probes before analyzing the array as they are likely to provide a noisy signal (Additional file 2). Or conversely, this could be considered rescuing a total of 122,253 of the potentially ‘noisy’ probes (7,367 Infinium I and 114,886 Infinium II, Table 1).

Our remaining recommended set of high-quality probes provides adequate coverage of the genome with only a select few regions being underrepresented. Figure S4 in Additional file 1 shows that only the HLA region on Chromosome 6 shows removal of 100% of the probes, with four other genomic regions showing greater than 60% removal of probes. When considering particular genomic features, in all categories except enhancers, the average fraction of probes removed is below the genome-wide average of 39% (Figure S5 in Additional file 1). This suggests that probe filtering will not affect the ability to call differential methylation in these regions. It also suggests that the bulk of probe filtering happens for probes elsewhere in the genome. However, when we look in detail at CpG islands (Figure S6 in Additional file 1), we see 47% of the probes designed to hybridize the N Shelf (region 2–4 kb upstream of CpG island) and S Shelf (region 2–4 kb downstream of CpG island) fall into our suggested removal set, thus reducing the ability to profile these regions. However, the island itself seems to have few potential ‘noisy’ probes on average (28%). At a gene level, if the genes are split into separate promoter and gene-body regions, 1,380 and 1,236 genes (out of a total of 21,235) have 100% of the probes fall into the filtered set respectively (Figure S7 in Additional file 1). This means that the interrogation of the methylation of some genes is not possible using our approach.

However, overall, these observations demonstrate that while we recommend 39% of the probes on the array should be removed from analysis, in most cases this will not significantly affect downstream genome-wide analyses. It is important to note however, that different filtering strategies may be adopted depending on the type of study being undertaken. The majority of the data analysed in this study has been derived from a single ethnic group of samples with white European/American descent. Therefore, information regarding filtering based on genomic variants would be specific to this ethnic group. Studies involving other ethnicities may adopt different filtering approaches to our recommended default. For instance, a study across diverse ethnic groups may need to adopt a more aggressive filtering approach, in absence of adequate genotyping information, as observed methylation changes may be more likely associated with genomic changes. However, if the study involved a homogenous ethnic population, a more relaxed filtering strategy may be adopted. Our filtering annotation (Additional files 2 and 3) provides information for users of the HM450K to make an informed decision given both of these study types.

## Conclusion

This study provides a comprehensive analysis of the effects of repeats, SNPs, INDELs, and reduced genome complexity on the performance of the Illumina HM450 bead methylation array. We show that a subset of probes on the array have the potential to provide a noisy methylation signal. We provide a principled way to identify and filter these probes. We also show that by applying this filtering procedure to primary data from the HM450 bead array, it is possible to yield analyses of significant interest from a biological perspective that may be unobservable without principled probe filtering.

## Methods

### Annotation of probes on the HM450K bead array

Probe annotation information including sequence and chromosome location for the IlluminaHumanMethylation450 array was obtained from [55] (humanMethylation450_15017482_v.1.2.csv). In addition, annotations from the R package: IlluminaHumanMethylation450probe ([56], containing 485,512 probes) were also extracted. For consistency, the probe sequences and their genomic strand information were compared between these two annotation sources. Some inconsistencies were observed with respect to the SourceSeq column reported in the table – the sequence in which the probe was derived. We used the human reference genome hg19 [57] to determine the correct strand and sequence annotation for each probe.

### Analysis of H1-hESC Whole Genome Bisulfite sequencing data, HM450K and genotyping from ENCODE

We obtained CpG methylation data (bigBed format) assayed by Whole Genome Bisulfite Sequencing (GEO GSE40832), and HM450K profiling (GEO GSE40699), as well as genotyping information (GEO GSM999275), for H1-hESC cells generated by the ENCODE production group [38]. We converted the bigBed files into ASCII bed files using the bigBedToBed method downloaded from the UCSC Genome Browser [57]. The bed files contained base-by-base methylation information including chromosomal location, and methylated score range from 0 to 100. In order to determine which probes on the HM450K bead array mapped to specific locations on targeted WGBS, each probe was searched against ‘single base chromosomal locations’ on WGBS data. If an occurrence of a certain base was found, the corresponding base was linked to the interrogated probe. For analysis, we considered only those sites which have at least 5 reads supporting the methylation status in the WGBS data.

### Calculating correlation between HM450K beta-values and WGBS

Figures 1a and 1b show the contour plots illustrating the relation between WGBS and HM450K bead array. The following method was used to generate these graphs. First, the 2-dimensional methylation beta values matrix (2DMM) was formed comprising WGBS and HM450K bead array on each dimension (range from 0 to 1). Second, the matrix was divided into consecutive sub-matrixes of window size 0.02 and the total number of probes in each sub-matrix was counted. The results have shown that majority of the probes lie at or nearby the (0,0) and (1,1) coordinates of the 2DMM. To illustrate this, the resulting 2DMM with counts was input into the R package to generate contour plots using the ‘filled.contour’ function [58].

### Analysis of HM450K Data of 63 samples from the ENCODE project

We obtained data for 63 samples (comprising 53 different cell-lines) profiled using the HM450K bead array from GEO, accession number GSE40699. Preprocessing and analysis of the HM450K bead array data available was performed using the minfi Bioconductor package [22] and (subset-quantile) SWAN normalization [21].

### Analysis of primary data from human prostate cancer samples

Patients undergoing radical prostatectomy for prostate cancer had fresh frozen samples of cancer as well as adjacent benign prostate tissue prospectively stored in a cancer biorepository program [59]. Institutional review board approval was granted and all patients consented to use of their de-identified tissue samples for genomic analysis (Melbourne Health Human Research and Ethics Committee, 2010.082). Four tumour samples containing Gleason 6 cancer and four benign samples from other prostate glands containing Gleason 6 cancer were selected for study. Tissue samples were cryosectioned for histopathological assessment. Genomic DNA was extracted from the homogenized samples using the Allprep Micro Kit (Qiagen, CA, USA) following manufacturer’s instructions and bisulfite converted using the Zymo EZ DNA Methylation kit (Zymo Research Corporation, CA, USA). The resulting libraries were hybridized onto the Illumina HumanMethylation450 BeadChip. Raw intensity data was generated using an iScan microarray reader (Illumina).

IDAT files were loaded into the R environment (2.15 development version) using the minfi package [22].We then preprocessed the data by converting the raw intensities (represented as Red and Green channel) into methylated and unmethylated signals applying the ‘MSet.raw’ function of minfi. Then, we applied the SWAN [21] function to normalise the data within the arrays.

To determine the differentially methylated patterns between (benign and tumour) samples, we applied the ‘dmpFinder’ minfi function. It uses the F-test to identify the differentially methylated sites between samples. The resulting P-values were multiple tested corrected using qvalue.cal function as described in the siggenes Bioconductor package [60] and a P-value of < 0.05 was applied as cutoff.

To map probes to genes, we downloaded gene coordinate information (refGene) from the UCSC Genome Browser database [57], which contains the chromosomal locations of 40,042 transcripts comprising 23,635 human genes. Probes were matched to the promoter region (from 1.5 kb upstream of the transcription start site (TSS)) and the body region (between TSS and transcription end site) of each transcript. First, we loaded the.

### Gene network construction

The following procedures were used to build the unfiltered, filtered and conservative filter analysis gene networks (Figure 2).

### Unfiltered analysis gene network

First, the differentially methylated probes (P < 0.05) were mapped to refGenes as described above. Then the resulting genes input into the String-String database to determine which genes were interact with each other, thus constructing the unfiltered analysis gene network.

### Filter analysis network

First, the recommended noisy probes (in total 190672 (75,292 Infinium I and 115,380 Infinium II)) were removed from the analysis pipeline. Second, the differentially methylated probes were determined and mapped to refGenes. Finally the derived gene list was input into the String-String database to identify the interacting genes, thus forming the filter analysis network.

### Conservative filter analysis

Initially, the potential noisy probes (319,545 or 65% of the probes, Table 1) were removed and then differentially methylated probes were determined and mapped to refGenes. Finally, the resulting gene list was input into a String-String database to form the conservative filter network of interacting genes.

### Analysis of blood samples

First, we obtained four blood samples comprising two males and two females profiled with HM450K bead array from the study of [29] (GEO GSE42409). Each sample contains the beta value for 428,216 probes. Secondly, we downloaded a matrix file (GEO GSM1002649) that contains the beta values for 473,034 probes on the HM450K bead array in blood samples of 656 individuals, aged 19 to 101 [54]. As described in [29] we also selected a subset of 261 individuals ranging in age 19 to 61. Finally, in both datasets the standard deviation (SD) for each probe has been calculated.

### Description of probe filtering procedures

Figure S8 in Additional file 1 provides a workflow of our probe filtering process which occurs prior to methylation status calling. Each step is outlined below:

1. **Determining probe uniqueness across genome:** All probes which hybridize multiple locations in the genome have the possibility of providing conflicting methylation calls. In order to determine which probes map to multiple locations, we generated a “bisulfite genome” whereby all C’s were converted to T’s. Probes called as unique by Novoalign bisulfite mode [61] to hg19 reference genome were identified. Any probes matching more than 1 genomic location were considered difficult to interpret and subsequently removed from further analysis [62].
2. **Does the probe map to repetitive sequence elements?** In order to determine which probes overlap with repetitive regions, the repeat-masked annotation files (generated using RepeatMasker) for every chromosome were downloaded from the UCSC hg19 genome browser [57]. The genomic location of each probe was scanned for the identification of repeat sequence elements (RSE). If an occurrence of a certain RSE was found, the corresponding probe was removed from further analysis.
3. **Does the probe map to DNA harboring an INDEL?** dbSNP v135 was used to identify known small insertions and deletions that overlapped probe hybridization locations in genome. Any probes hybridizing across known INDELs were kept for subsequent analysis (see result section for details).
4. **Does the probe map to DNA containing a SNP?** dbSNP v135 [63] was used to identify all known single nucleotide polymorphisms. If the probe target sequence contained no known SNPs, the probe was retained for further analysis. If the probe target sequence contained a SNP, the probe was subjected to further filtering analysis (steps 5–6) before deciding if this probe was kept for analysis or removed.
5. **Does the probe map to sequence with a SNP at the interrogated CpG?** If a known SNP was located at the target CpG site, the probe was removed from further analysis.
6. **Is the SNP in the probe OK in bisulfite space?** The majority of C’s in the genome that are not followed by a G are likely to be unmethylated. Therefore, when bisulfite treated, these C’s will appear as T’s in the genome and corresponding probe sequences [2]. Given this, if a particular SNP causes a C– > T or T- > C change, it will always be observed as a T in a bisulfite treated genome. Therefore, there are a number of probes which are not affected by these SNPs as they are OK in bisulfite space. To find these, we considered all C– > T and T- > C SNPs and observed their neighbouring base downstream. If the base was not a G, these SNPs were considered OK in bisulfite space and their effects were ignored.

## Electronic supplementary material

Additional file 1: **Contains supplementary Figures S1-S10.** (PDF 2 MB)

Additional file 2: **A table containing all filtering information.** (CSV 16 MB)

Additional file 3: **Contains a description of the column headers for Additional file** 2 **.** (PDF 262 KB)

## Abbreviations

HM450K bead array: HumanMethylation450
WGBS: Whole-genome bisulfite sequencing
HM27K array: HumanMethylation27
SNPs: Single nucleotide polymorphisms
INDELS: small insertions and deletions
CpGs: cytosines that are followed by guanines
MeDIP-seq: methylated DNA immunoprecipation sequencing
RRBS: reduced representation bisulfite sequencing
MethylCap-seq: methylated DNA captured by affinity purification.
