# Supplementary material for: Reducing the risk of false discovery enabling identification of biologically significant genome-wide methylation status using the HumanMethylation450 array
Source: BMC Genomics. 2014 Jan 22;15(1):51. doi: 10.1186/1471-2164-15-51 (PMC3943510; doi:10.1186/1471-2164-15-51)
Supplement: Supplementary file 1 — Additional file 1: Contains supplementary Figures S1-S10. (PDF 2 MB) [file 12864_2013_7006_MOESM1_ESM.pdf]

Supplementary Material

Reducing the risk of false discovery enabling identification of biologically significant genome-wide methylation status using the HumanMethylation450 array

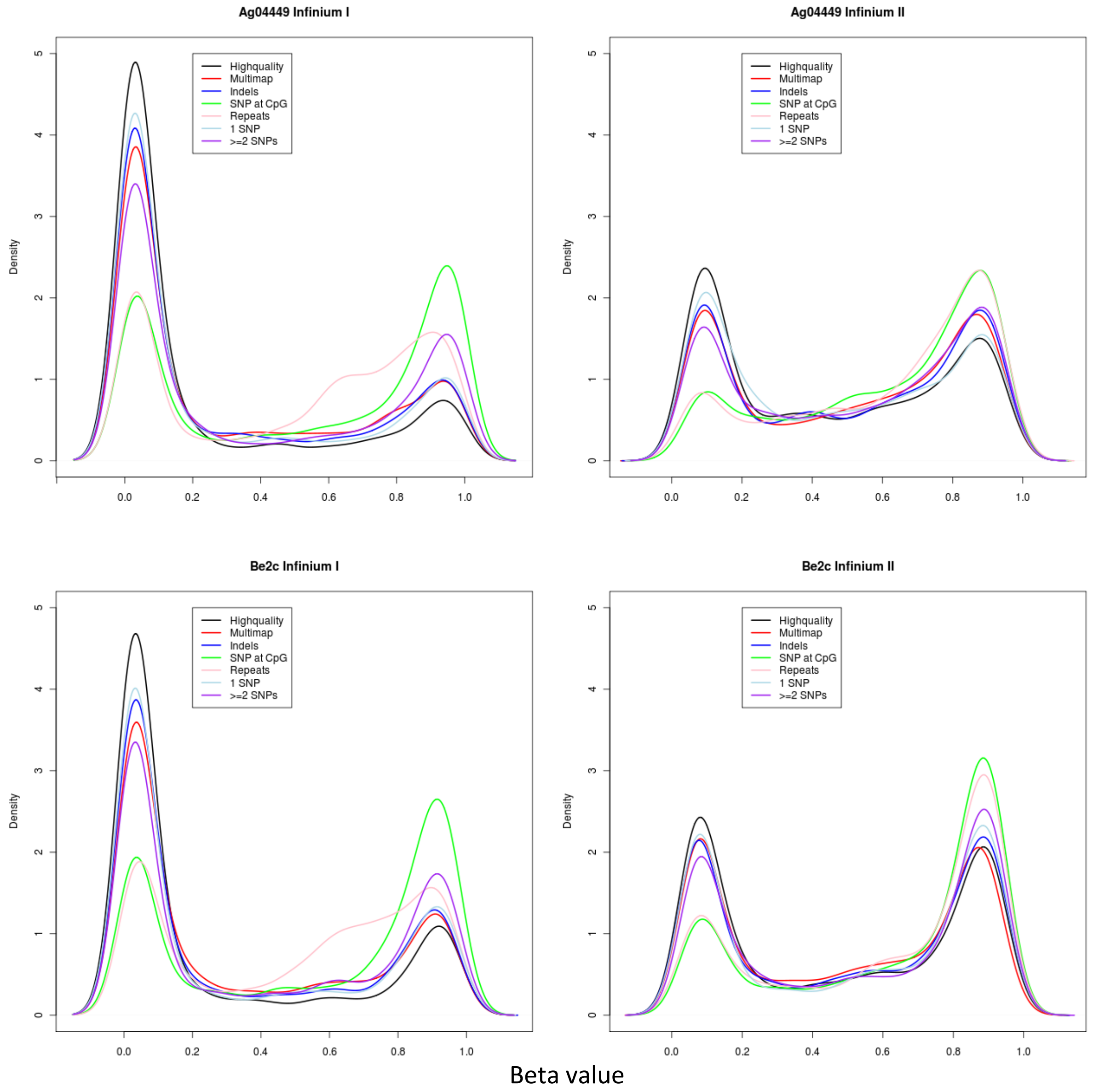

Figure S1. Shows density distributions for beta value for different sets of probes. In each graph, a high-quality or set of probes is plotted in black. Density plots for two ENCODE cell lines are shown for Infinium I and Infinium II probes. All 63 ENCODE samples show the same trend as the above samples (data not shown). The area under each curve has been normalized to total to 1, even though different numbers of observations are in each category.

a

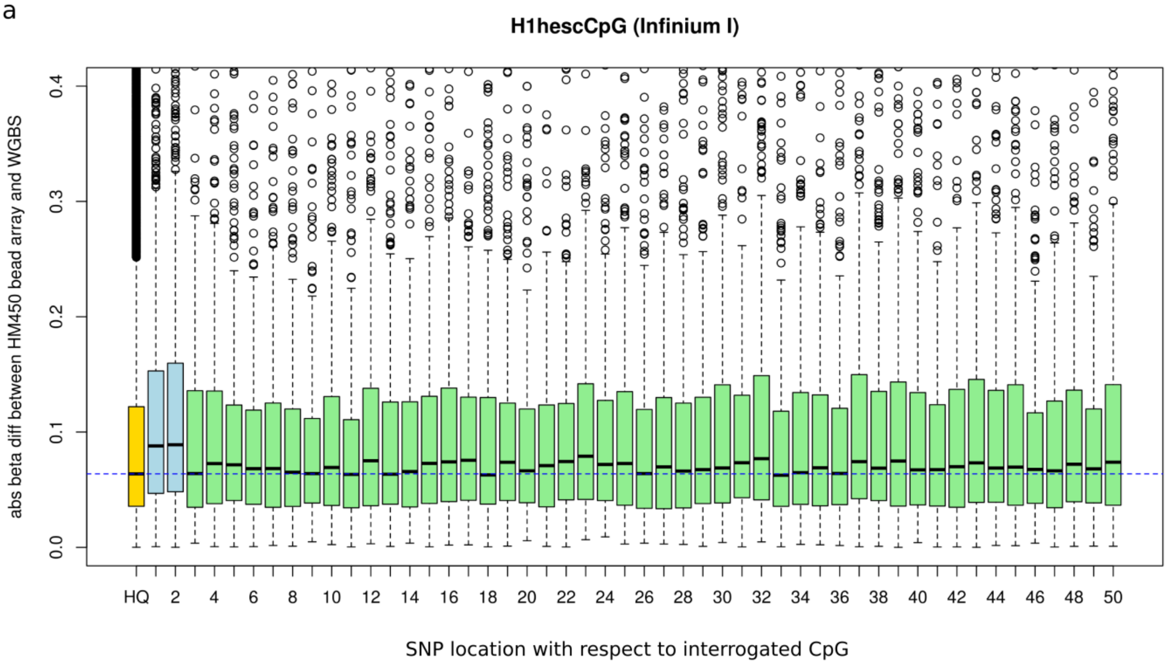

b

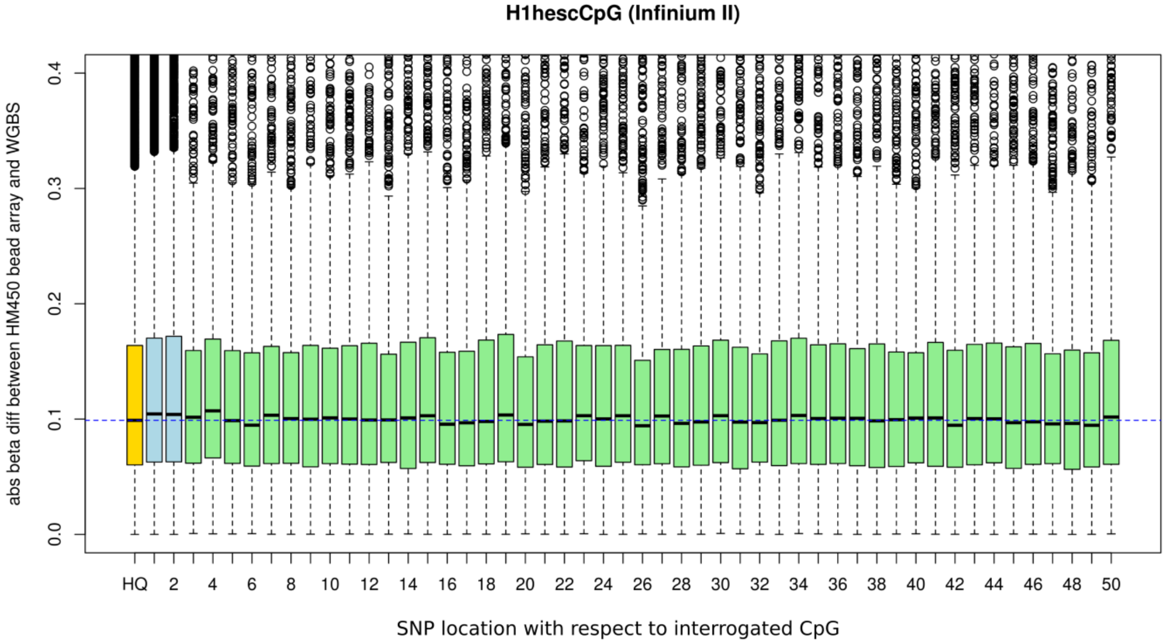

Figure S2. This figure plots the absolute beta difference (compared to the high-quality probe set in gold) for probes which have SNPs at a particular distance from the interrogated CpG (x-axis) for Infinium I probes (a) and Infinium II probes (b).

a

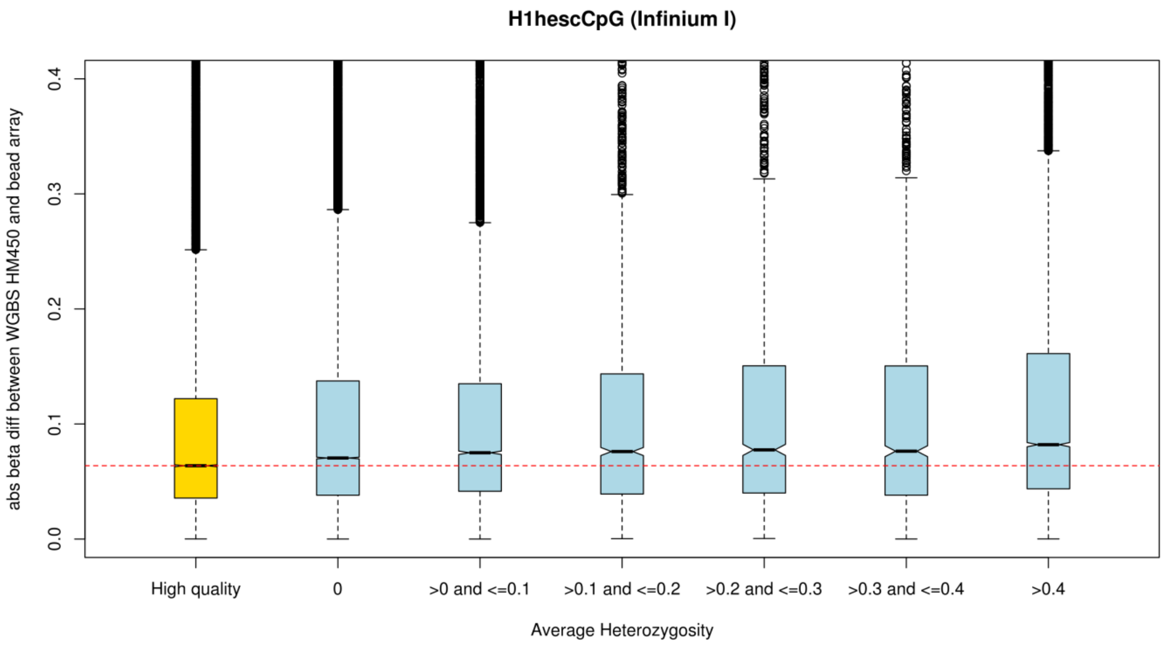

b

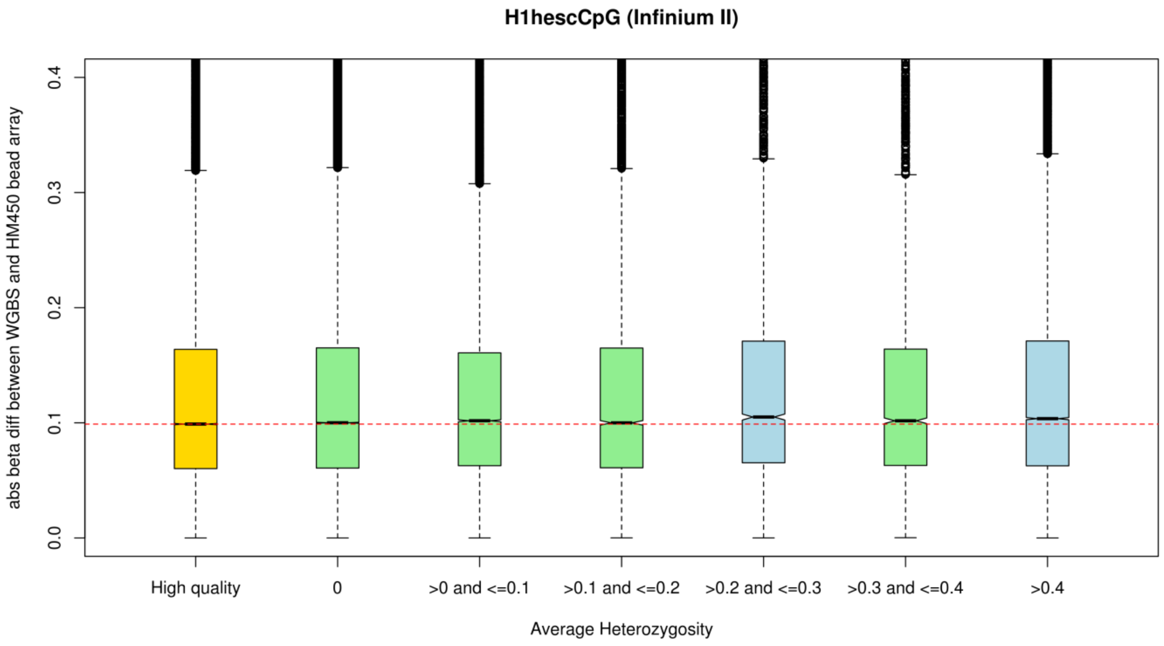

Figure S3. This figure plots the absolute beta difference (compared to the high-quality probe set in gold) for probes which have SNPs with varying levels of average heterozygosity (x-axis) for Infinium I probes (a) and Infinium II probes (b).

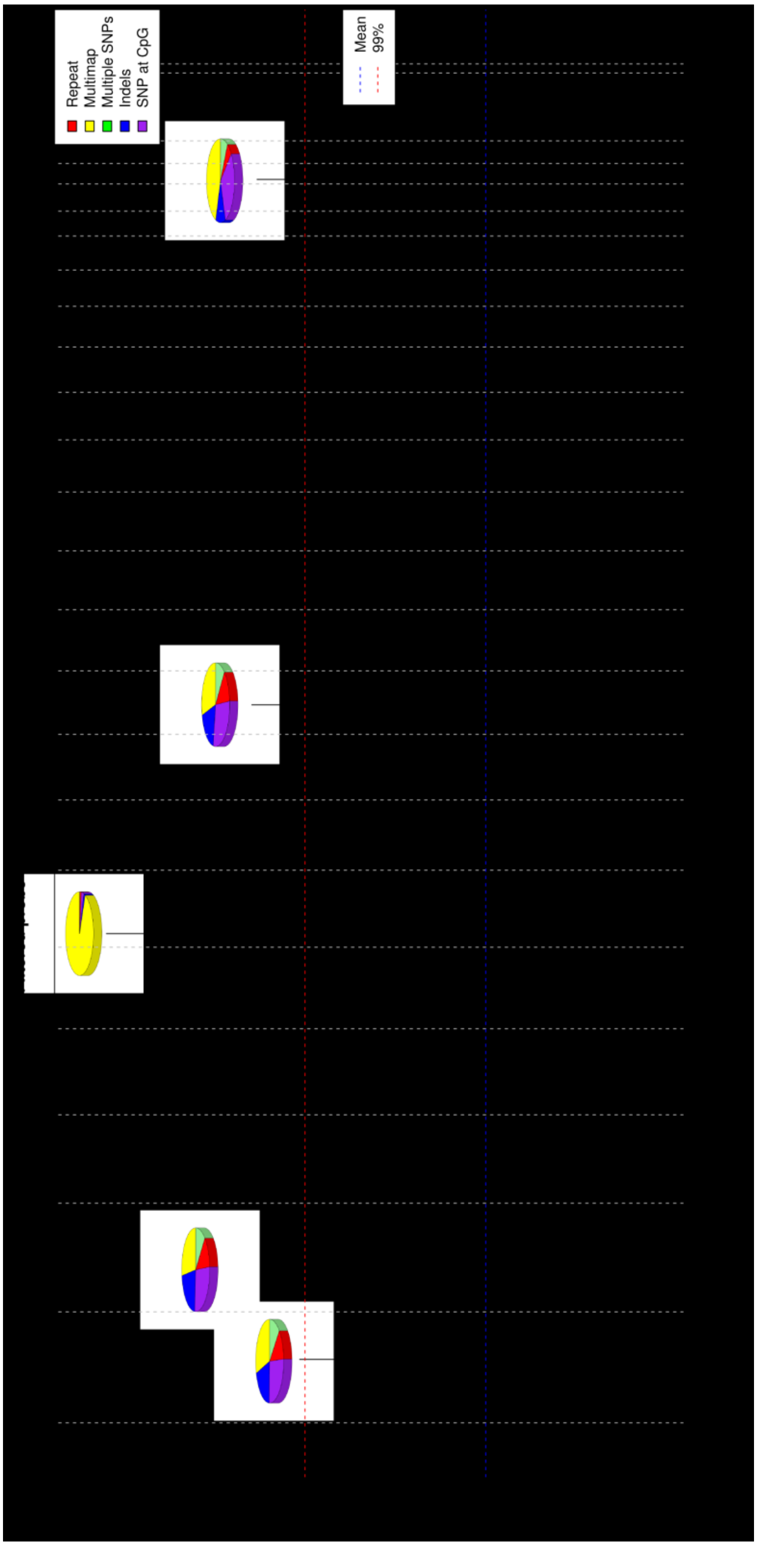

Figure S2. A plot showing the distribution of probes predicted to be "noisy" across the genome. Probes were counted for each 6MB window across the genome and the fraction of filtered probes was plotted. The coloured dotted lines show the mean (green), 99 percentile (red) For regions of the genome which have a larger than expected number of filtered probes, a pie chart is given showing the categories of filtering.

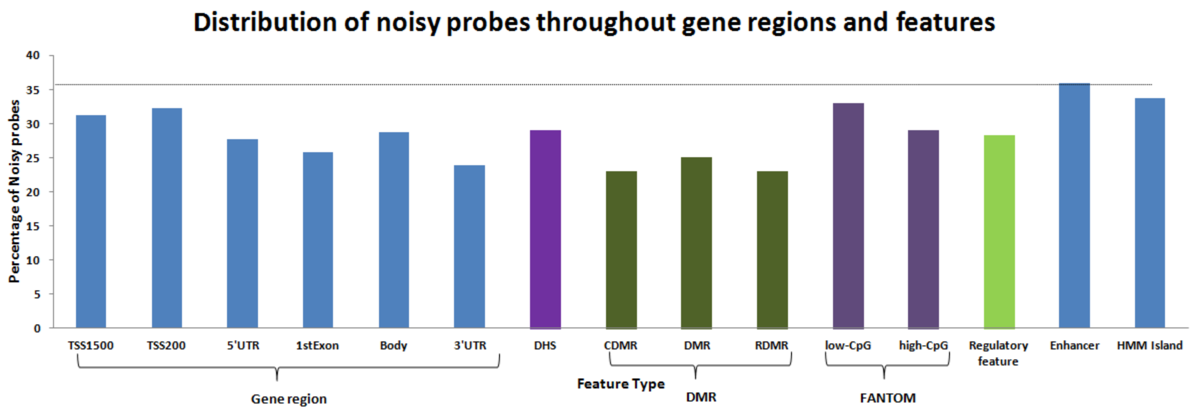

Figure S5. This figure shows for varying genomic features, the percentage of probes which are filtered.

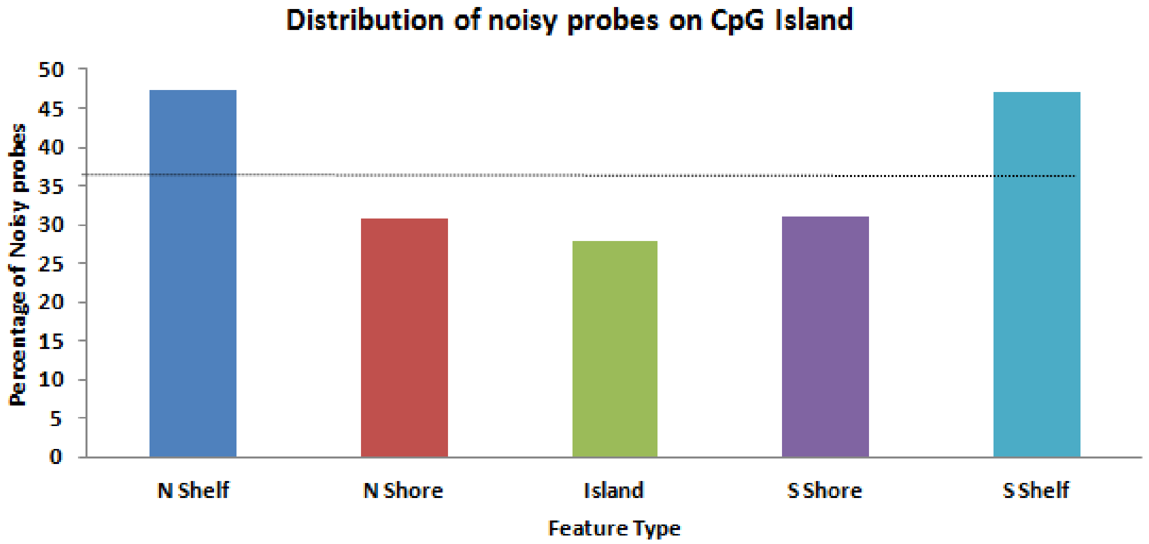

Figure S6. This figure shows the percentage of probes that we recommend removing for different features in a CpG Island The N shelf is defined as region 2-4 kb bp upstream of CpG island, N Shore is 0-2 kb bp upstream of CpG Island, S Shore is 0-2 kb downstream of CpG island and S Shelf is 2-4 kb bp downstream of CpG island.

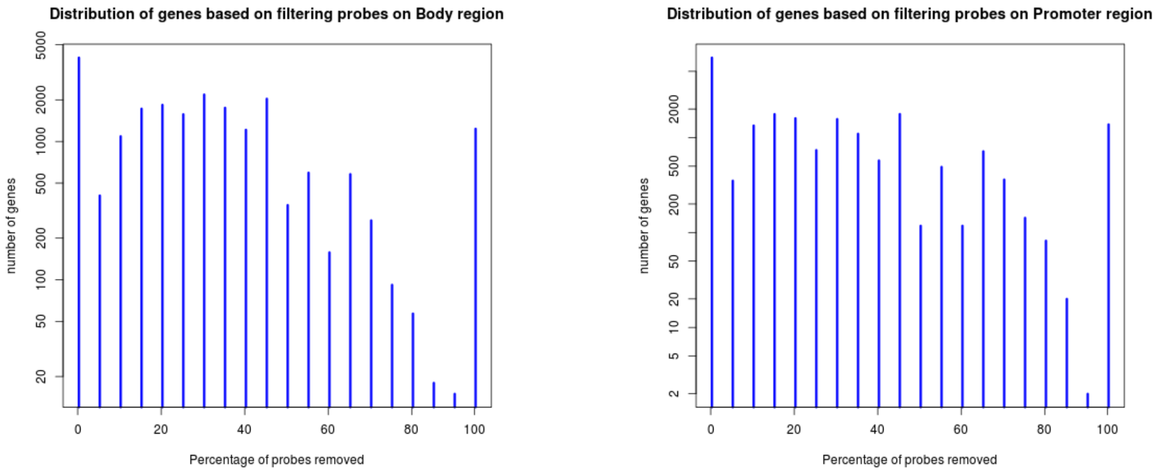

Figure S7. This figure shows the number of genes (y-axis) which have a certain percentage of probes covering the gene removed by our filtering procedure (x-axis). On the left we consider gene bodies and on the right we consider gene promoters.

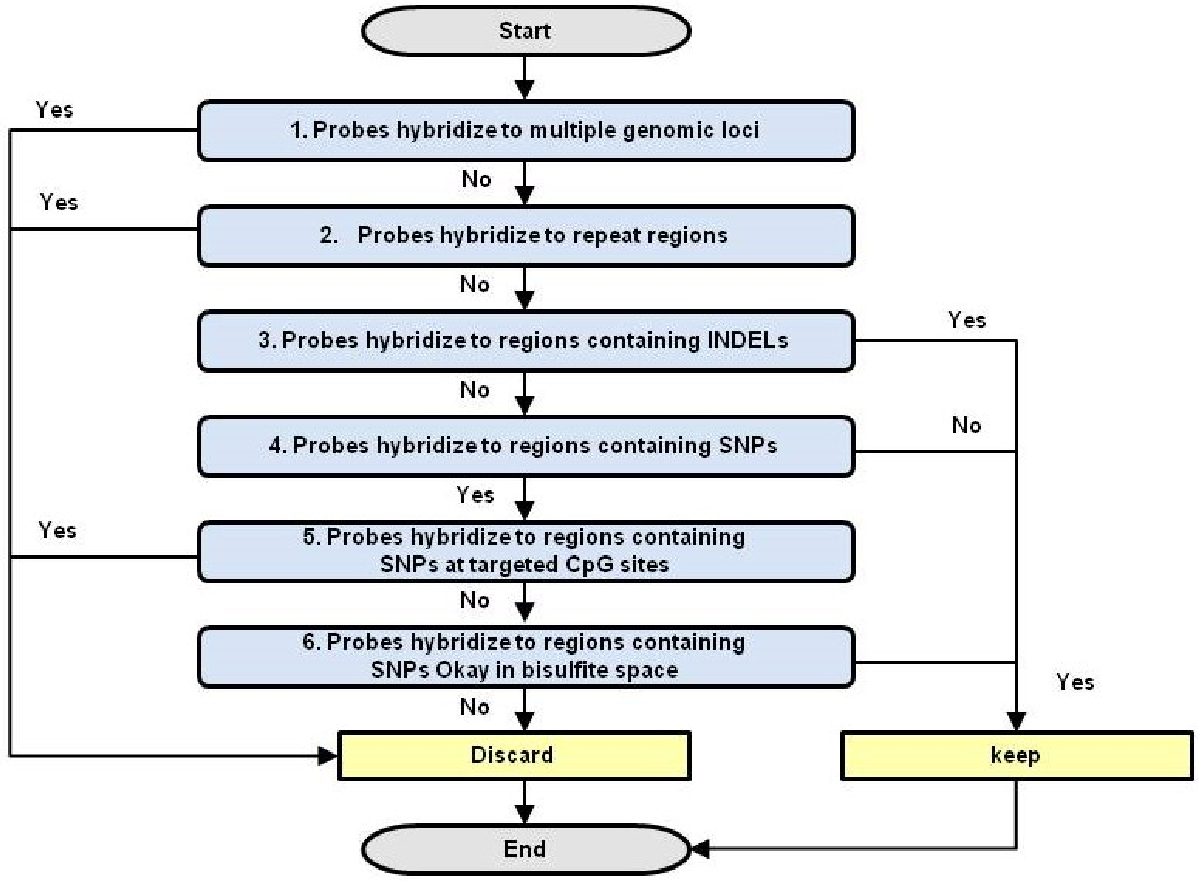

Figure S8. Workflow for determining affected Probes. In step (1) the probes which were not aligned to unique, unambiguous loci in the human genome were discarded. In step (2), probes which hybridize to regions overlapped with repetitive DNA regions were discarded. In step (3), probes which hybridize to regions containing only known indels that is, they represent the insertion or deletion of one or multiple nucleotides were kept for the subsequent analysis. Next (step 4), the SNP-containing probes have been marked and subjected for downstream filtering analysis (Steps (5-6)). First, the probes which hybridize to regions containing known SNP at the interrogated CpG sites were discarded (step 5). Then, the probes which were unlikely to be affected by those SNPs that were okay in bisulphite-treated genome space (step 6), kept for further analysis.

(a)

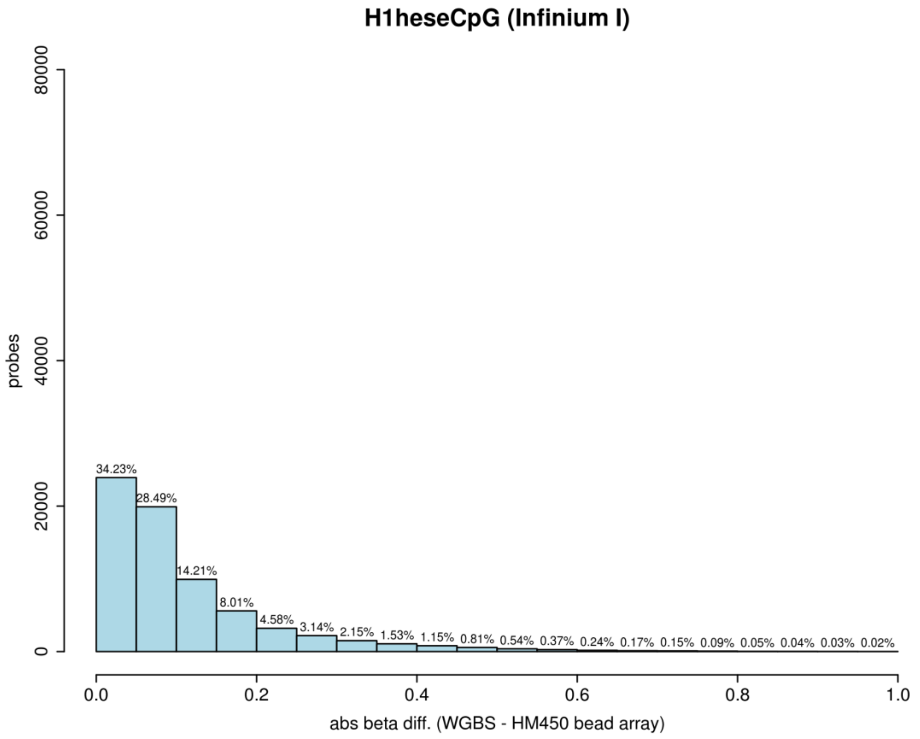

(b)

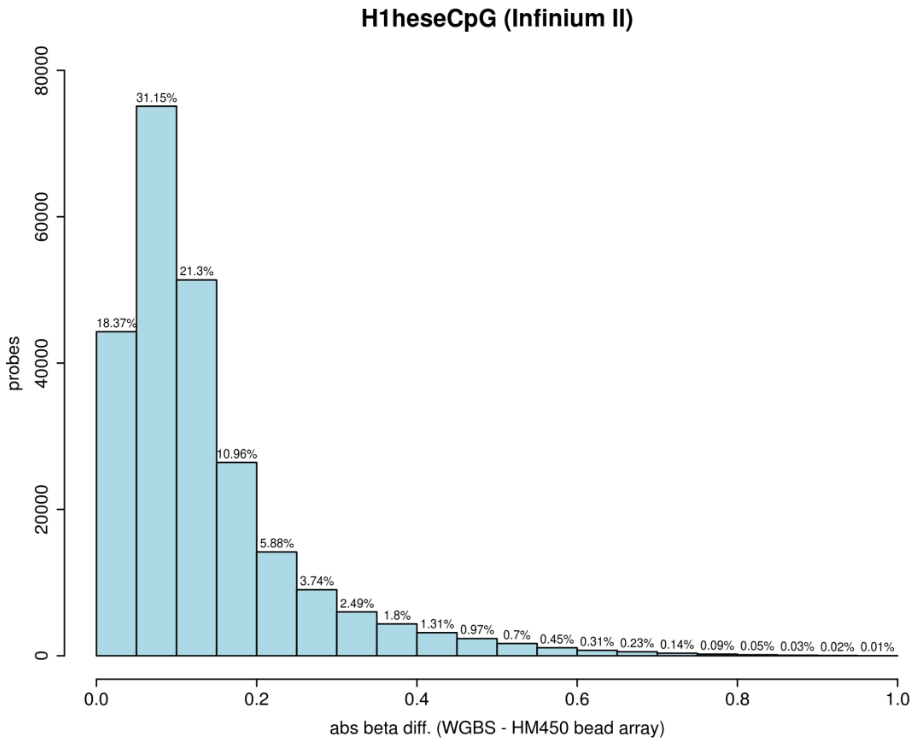

Figure S9. (a) and (b) show the histogram of the number of probes (x-axis) based on their absolute beta differences between WGBS and HM450 bead array (y-axis), plotted separately for Infinium I and Infinium II probes. Each histogram bar is labeled with the percentage of probes lie in a given bin.

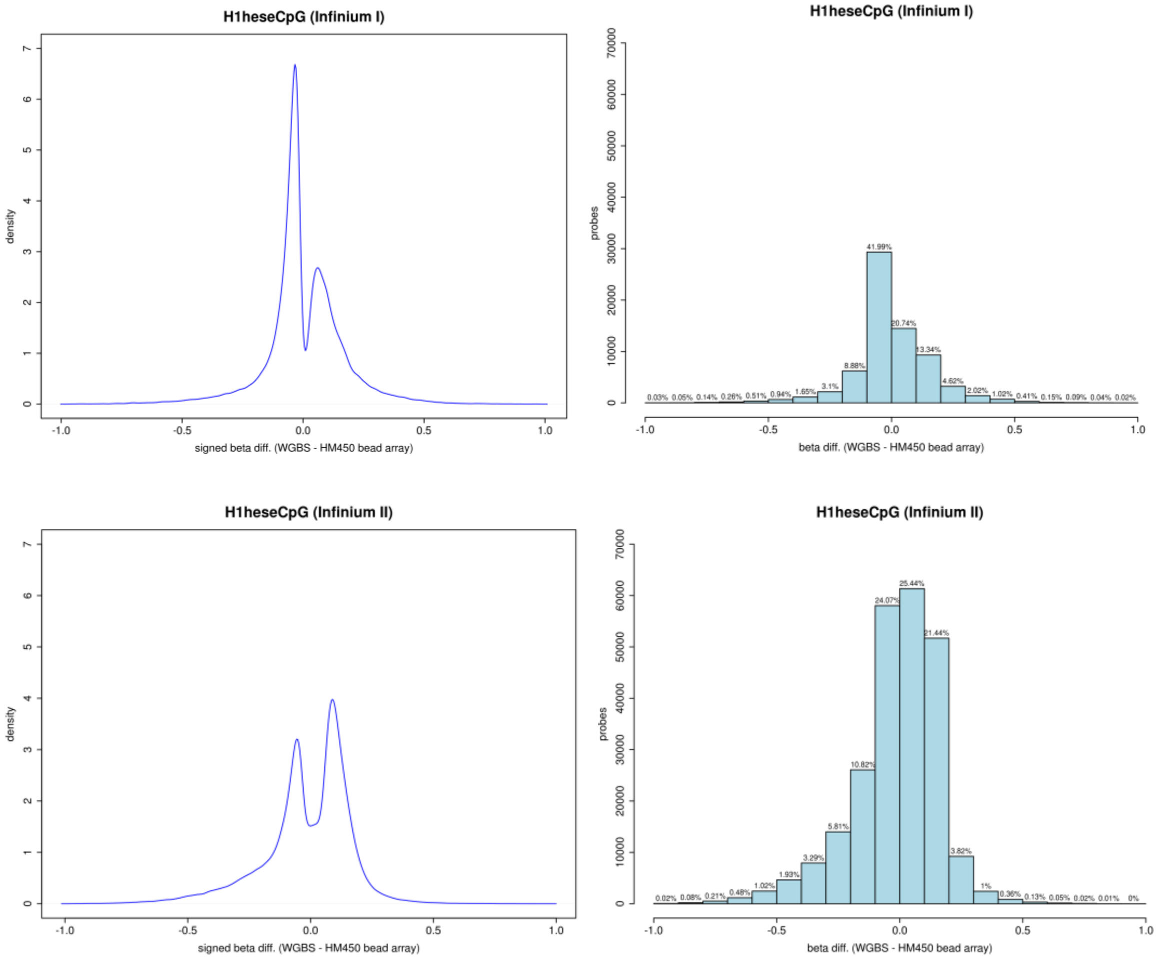

Figure S10: (a) and (b) show the density as well as histogram of the number of probes based on their signed beta differenences (y-axis) between WGBS and HM450K bead array, plotted for Infinium I and Infinium II probes using H1heseCpG dataset.
