# Supplementary material for: Reducing the risk of false discovery enabling identification of biologically significant genome-wide methylation status using the HumanMethylation450 array
Source: BMC Genomics. 2014 Jan 22;15(1):51. doi: 10.1186/1471-2164-15-51 (PMC3943510; doi:10.1186/1471-2164-15-51)
Supplement: Supplementary file 3 — Additional file 3: Contains a description of the column headers for Additional file 2 . (PDF 262 KB) [file 12864_2013_7006_MOESM3_ESM.pdf]

This file contains brief description for the supplementary file (Additional file 2.csv) file columns:

1. **Probe**
This column contains the probe ID as provided by Illumina.

2. **SNP+INDEL_count**
This column gives the total number of known SNPs (dbSNP ver135) and INDELs overlapping the probe region. Multiple SNPs that mapped to same location counted as 1.

3. **Flag(discard/keep)**
This column contains the word ‘discard’ or ‘keep’. The word “**keep**” indicates that the given probe is recommended for subsequent analysis, whereas the word “**discard**” means that the probe is recommended to be removed from the subsequent analysis.

4. **MultiMap**
This column contains value of either 1 or 0. The value of “1” indicates that the corresponding probe hybridizes to multiple genomic loci whereas the value of “0” represents that the probe maps to unique genomic loci.

5. **Indels**
This column provides the total number of INDELs which reside in the region hybridized by the probe.

6. **SNP-at-CpG**
This column holds value of either 1 or 0. The value of “1” indicates that the given probe mapped to sequence with the SNP at the interrogated CpG whereas the value of "0" represents that the probe does not contain any SNP at the interrogated CpG site.

7. **Repeat**
This column contains the value of either 1 or 0. The value of “1” illustrates that the probe spans a region in the genome containing repeat sequence elements whereas the value of “0” represents that the probe hybridizes to non-repeat regions.

8. **WGBS_HM450K_GT_0.3**
This column contains the value of either 1 or 0. The value of 1 represents that, for a given probe, the absolute beta difference between whole-genome bisulfite sequencing (WGBS) and Illumina HumanMethylation450 (HM450K) bead array is greater than 0.3 while the value of 0 shows that the probe holds absolute beta difference (WGBS-HM450K) less than or equal to 0.3.

9. **ProbeType**
This column contains the character(s) I or II. The character ‘I’ indicates that the probe is of Infinium I type where as a character of ‘II’ indicates that the given probe is of Infinium II type.

10. **BisOK**
This column provides the total number of SNPs that were okay in bisulfite-treated genome.
